# A new procedure to analyze polymorphonuclear myeloid derived suppressor cells in cryopreserved samples cells by flow cytometry

**DOI:** 10.1371/journal.pone.0202920

**Published:** 2018-08-30

**Authors:** Alessandra Sacchi, Nicola Tumino, Germana Grassi, Rita Casetti, Eleonora Cimini, Veronica Bordoni, Adriana Ammassari, Andrea Antinori, Chiara Agrati

**Affiliations:** 1 Cellular Immunology and Pharmacology Laboratory, “Lazzaro Spallanzani” National Institute for Infectious Diseases, IRCCS, Rome, Italy; 2 Clinical Department, “Lazzaro Spallanzani” National Institute for Infectious Diseases, IRCCS, Rome, Italy; Universidade Federal de Sao Paulo, BRAZIL

## Abstract

Myeloid derived suppressor cells (MDSC) is a heterogeneous subset of immature and mature cells of the myeloid lineage, undergoing expansion during pathologic conditions, and able to perform strong immune suppressive functions. It has been shown that cryopreservation selectively impacts the polimorphonuclear (PMN) MDSC viability and recovery, and alters the correct analysis of MDSC subsets. In laboratory practice, cryopreservation is often inevitable, in particular in multicenter studies where samples have to be shipped to a centralized laboratory. Aim of the present work was to set out a new protocol to evaluate the frequency of PMN-MDSC in thawed cells by flow-cytometry. PBMC were isolated from HIV+ patients and healthy donors, and were cryopreserved for at least ten days. After thawing, two different protocols were used: 1. standard protocol (SP) consisting of staining with the antibodies mix and then fixing with formalin 1%; 2. thawed protocol (TP) in which fixation foregoes the staining with the antibodies mix. Results showed that PMN-MDSC frequency in *ex vivo* PBMC evaluated by means TP was comparable to that analysed by SP, indicating that the protocol did not alter PMN-MDSC quantification in *ex vivo* cells. We then demonstrated that PMN-MDSC frequency in thawed PBMC tested by TP was almost identical to the frequency obtained in *ex vivo* cells evaluated by using SP. However, we observed that after three hours of culture post-thawing, PMN-MDSC were not assessable anymore with both SP and TP. In conclusion, we herein demonstrated that fixing PBMC soon after thawing and before antibody staining allows preservation of PMN-MDSC integrity and a reliable cells quantification. Thus, it is possible to phenotipically identify PMN-MDSC in cryopreserved PBMC, consenting adequate test precision and accuracy as well as making multicentre research more feasible.

## Introduction

The activation of the immune system is finely regulated by many different mechanisms and cell subsets, such as regulatory T cells and myeloid derived suppressor cells (MDSC). MDSC are a heterogeneous cell population comprising myeloid cell progenitors and mature cells [1]. In pathological conditions, such as cancers, different infectious diseases, or some autoimmune disorders, a partial block in the differentiation of immature into mature myeloid cells results in the expansion of MDSC population [2]. MDSC have been originally discovered in mice, and then shown in patients with tumours by numerous studies [3–7]. Recently, MDSC were also found in peripheral blood of patients with infectious diseases, namely HIV [8–10] and HCV infection [11], as well as tuberculosis [12]. Their presence in the tumour tissues or in peripheral circulation has been associated with poor prognosis, and the use of MDSC as a predictive biomarker for the clinical outcome following oncologic treatment is under evaluation [13, 14]. A correlation between MDSC frequency and disease progression has been also demonstrated for HIV infection [10, 15]. MDSC are currently under extensive investigation, and numerous clinical and laboratory studies are in progress to determine the role of MDSC in oncologic and infectious diseases progression. MDSC were described in mice as cells expressing CD11b and Gr-1 and capable of strong immune suppressive functions. Subsequent studies showed that CD11b^+^/Gr-1^+^ cells are not a homogeneous cell population, but comprise at least two main subsets, the monocytic (Mo-MDSC) and the polymorphonuclear (PMN-MDSC) subsets, and that these cells can be identified by evaluating different markers. In particular, in mice the differential expression of the two isoforms of Gr-1 (Ly6C and Ly6G), allows the identification of PMN-MDSC and Mo-MDSC as CD11b^+^/Gr-1^high^/Ly6C^−^/Ly6G^high^ and CD11b^+^/Gr-1^int^/Ly6C^high^/Ly6G^−^ respectively [16].

Human MDSC do not express Gr-1, and until recently, because of the lack of a specific molecule characterizing these cells, the markers used to identify human MDSC by flow cytometry were largely heterogeneous. Moreover, PMN-MDSC are phenotipically indistinguishable from granulocytes, and it is possible to identify PMN-MDSC only after mononuclear cells isolation. In fact PMN-MDSC, differently from the other granulocytes, stratify with mononuclear cells after gradient centrifugation [17]. Bronte and colleagues very recently proposed a recommendation for the definition of MDSC in PBMC from humans. In particular, they indicate the minimal phenotypic characteristics to identify MDSC, that is CD14^-^CD11b^+^CD15^+^(or CD66^+^) for PMN-MDSC and CD11b^+^CD14^+^HLA-DRl^ow/-^ CD15^-^ for Mo-MDSC [18]. Since the ability to suppress immune cells is a main feature of MDSC, in the same paper the authors defined the functional characteristics necessary to identify cells as MDSC, such as inhibition of anti-CD3/CD28 (or PHA) induced T-cell proliferation, or IFN-γ production by the addition of MDSC, or improved T-cell proliferation after removal of MDSC populations.

MDSC are very sensitive to some manipulation: it has been demonstrated that both, delay in sample processing [19] as well as cryopreservation [20] might have a negative impact on the viability of MDSC. In particular, while tolerating freezing/thawing, Mo-MDSC should not be stored for more than 4 h after blood drawn; instead PMN-MDSC may tolerate storage up to 26 h after blood drawn but are very sensitive to thawing. In laboratory practice cryopreservation is often inevitable, in particular in multicenter studies where samples have to be shipped to a centralized laboratory.

Aim of the present work was to set out a protocol to evaluate the frequencies of PMN-MDSC in thawed cells.

## Materials and methods

### Study group

Patients with HIV infection (n = 22) observed at the National Institute for Infectious Diseases (INMI) “Lazzaro Spallanzani” (Rome, Italy) were enrolled in this study. Sixteen healthy donors were used as control.

The study was approved by the Institutional Review Board of the INMI “Lazzaro Spallanzani” (ALPHA and SIREA studies) and written signed informed consent was obtained from all patients.

### PBMC separation

In order to get a high percentage of PMN-MDSC, peripheral blood mononuclear cells (PBMC) were isolated from peripheral blood of HIV+ patients by density gradient centrifugation (Lympholyte-H; Cederlane, Canada) within 4h from blood collection. After separation, PBMC were resuspended in RPMI 1640 (EuroClone, Italy) supplemented with 10% heat-inactivated fetal bovine serum (FBS) (EuroClone, Italy), 2 mmol/L L-glutamine, 10 mmol/L HEPES buffer (N-2-hydroxyethylpiperazine-N-2-ethane sulfonic acid) and with 2 mmol/L penicillin, and 50 μg/mL streptomycin (EuroClone, Italy).

### Cryopreservation and thawing procedures

5–10*10^6^ PBMC were firstly washed in PBS and then resuspendend in 250–500 μl respectively of cold freezing medium containing 90% heat-inactivated fetal bovine serum (FBS) (Euroclone) and 10% dimethylsulfoxide (DMSO) (Carlo Erba), dispensed in 2 ml cryovials (Euroclone). Samples were stored at -80°C for 10–90 days. Cells were thawed using the following procedure: cryovials were removed from -80°C and transferred to the 37°C water bath for thawing. Cells were then transferred drop by drop in 50 ml of pre-warmed at room temperature RPMI 1640 (EuroClone, Italy) supplemented with 10% heat-inactivated fetal bovine serum (FBS) (EuroClone, Italy), 2 mmol/L L-glutamine, 10 mmol/L HEPES buffer (N-2-hydroxyethylpiperazine-N-2-ethane sulfonic acid), 2 mmol/L penicillin, and 50 μg/mL streptomycin (EuroClone, Italy), and washed at 1000 rpm for 10 minutes. Cells viability was performed by trypan blue exclusion (viability 90%, data not shown) and immediately stained with the protocols described below. Alternatively, PBMC were cultured for 3 h in the above described medium.

### Staining procedures for flow cytometry analysis

Two different protocols were used to label fresh and thawed PBMC:

1. Standard Protocol (SP), consisted in washing PBMC in staining buffer (phosphate buffered saline-Bovine Serum Albumin-Sodium-NaN_3_, PBS-BSA-NaN_3_) and then incubating cells with antibodies for membrane staining at 4°C for 15 min in the dark. After washing with staining buffer, cells were fixed with 1% formalin (Sigma-Aldrich) for 10 minutes.
2. Thawed Protocol (TP), consisted in washing PBMC with PBS and then fixing with 1% formalin (Sigma-Aldrich) for 10 minutes. After one wash with staining buffer, cells were labelled with antibodies for membrane staining at 4°C for 15 min in the dark. Afterwards, cells were washed with staining buffer.

### Antibodies panel and flow cytometry

Evaluation of the MDSC percentage was accomplished with 0.5*10^6^ cells stained with FITC-conjugated anti-CD15, PE-conjugated anti-CD33, PerCP-conjugated anti-HLA-DR, a cocktail of APC-conjugated antibodies anti-CD3, -CD56, -CD19 (Lin), APC-H7-conjugated anti-CD14 and PE-Cy7-conjugated anti-CD11b (BD Biosciences). Acquisition of 100,000 events was performed in the leukocyte-gated population on FACS CANTO II and analyzed with FACS DIVA software (BD Biosciences, USA).

### Statistical analysis

GraphPad Prism version 4.00 for Windows (GraphPad Software, USA) was used to perform statistical analyses. The different protocols were compared using the paired Wilcoxon test and correlations were evaluated with the non-parametric Spearman test. Subjects groups were compared using the Mann Whitney test. A p value <0.05 was considered statistically significant.

## Results

### SP underrates PMN-MDSC frequency in thawed samples

**Fig 1:**
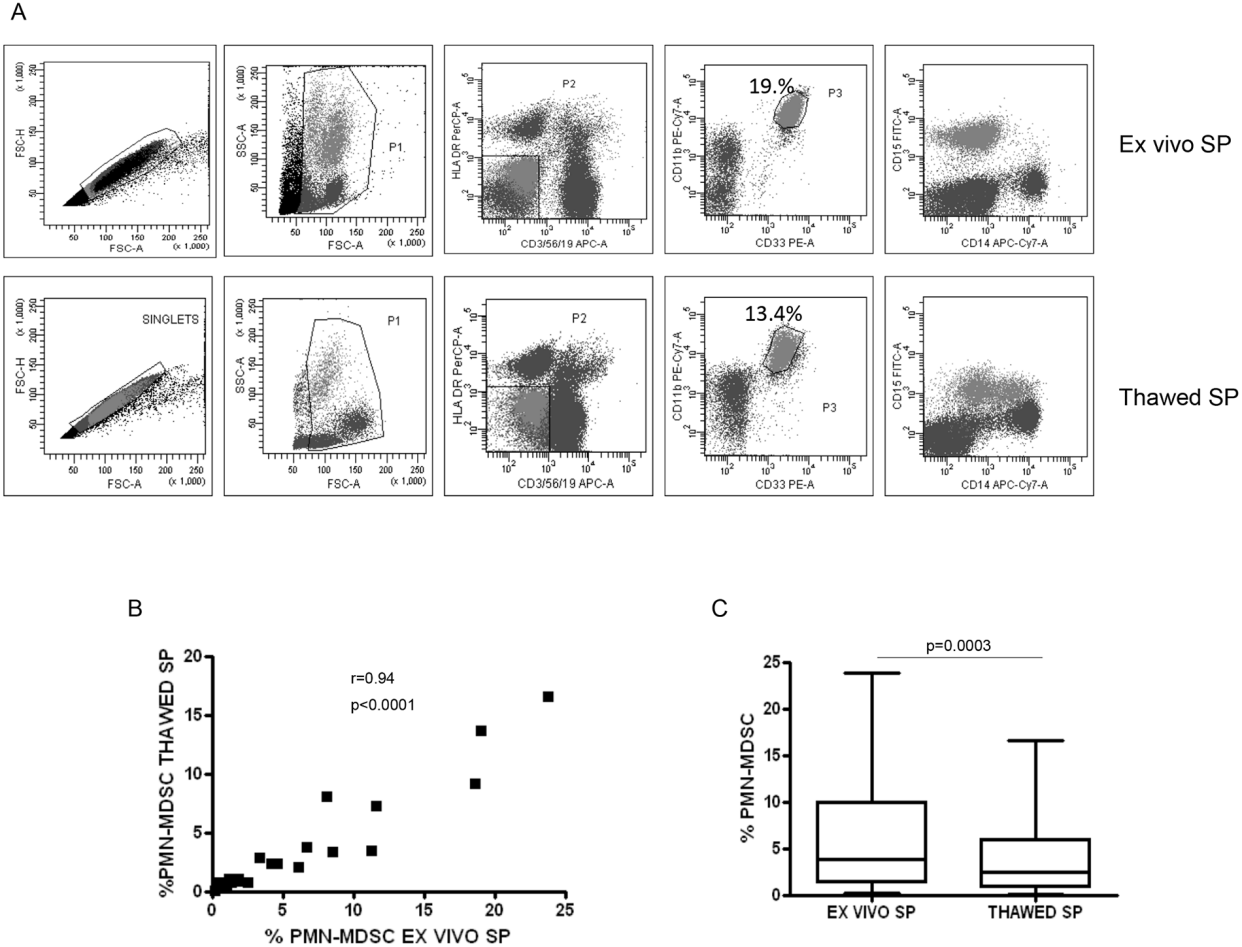
PMN-MDSC decreased after thawing. A. Gating strategy used to identify MDSC in *ex vivo* and thawed PBMC: after doublets exclusion (FSC-A/FSC-H), in the morphological gate (FSC/SSC) we excluded debris, then we gated Lin^-^/HLA DR^low/-^ cells. In this gate we selected CD11b^+^CD33^+^ cells (MDSC). The expression of CD14 and CD15 is shown on cells selected from the CD11b^+^CD33^+^ gate, to discriminate PMN-MDSC and Mo-MDSC. B. PMN-MDSC frequency in *ex vivo* and thawed PBMC from 22 HIV infected patients. Results are shown as Box and Whiskers. The Wilcoxon signed rank test was applied. C. Correlation between PMN-MDSC frequency in *ex vivo* and thawed PBMC was evaluated by the Spearman test. A p-value <0.05 was considered statistically significant.

We wondered whether the SP procedure on thawed PBMC underestimated PMN-MDSC as previously reported [20]. *Ex vivo* and cryopreserved (thawed) PBMC of 22 HIV+ patients were analyzed by flow cytometry and MDSC were identified as Lin^-^ HLA-DR^low/-^ CD11b^+^ CD33^+^. Moreover, the expression of CD14 and CD15 were used to distinguish Mo-MDSC and PMN-MDSC respectively. Fig 1A shows a representative example of the gating strategy, confirming the loss of PMN-MDSC after thawing [20] using the SP. In fact, although a good correlation has been observed (Fig 1B), a slight, but significant, reduction of the PMN-MDSC frequency after thawing compared with ex vivo PBMC (Fig 1C). Moreover, the SP performed on thawed cells gave rise to an abnormal CD14 expression (Fig 1A), indicating that freeze and thawing may alter PMN-MDSC phenotype.

### TP is comparable to SP in the identification of MDSC in *ex vivo* PBMC

**Fig 2:**
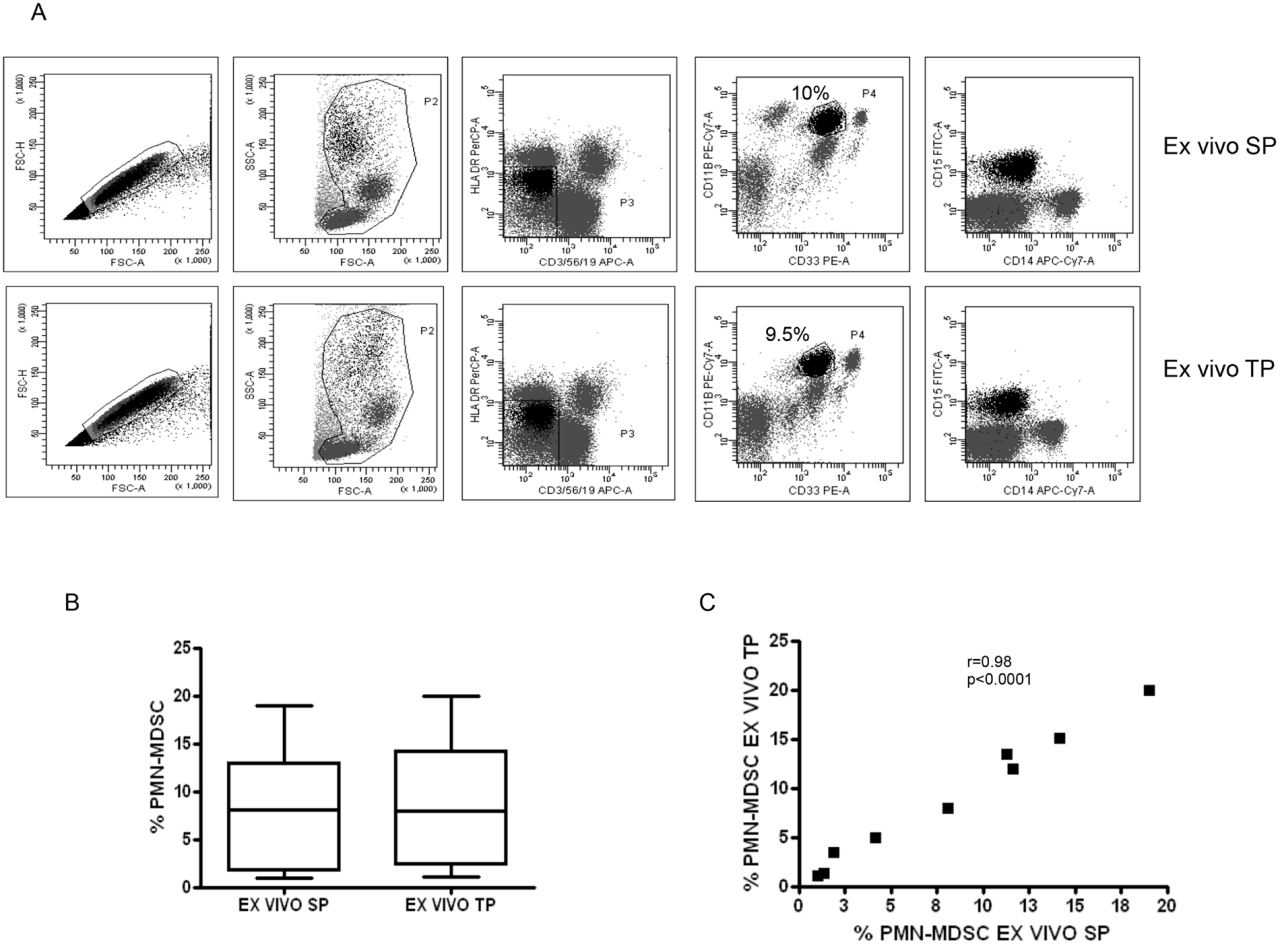
SP and TP are comparable in evaluating PMN-MDSC frequency in *ex vivo* PBMC. A. Gating strategy used to identify PMN-MDSC in *ex vivo* PBMC using SP and TP protocols: after doublets exclusion (FSC-A/FSC-H), in the morphological gate (FSC/SSC) we excluded debris, then we gated Lin^-^/HLA DR^low/-^ cells. In this gate we selected CD11b^+^CD33^+^ cells (MDSC). The expression of CD14, and CD15 is shown on cells selected from the CD11b^+^CD33^+^ gate, to discriminate PMN-MDSC and Mo-MDSC. B. PMN-MDSC frequency in *ex vivo* PBMC from 9 HIV-infected patients was evaluated by utilizing SP and TP. Results are shown as Box and Whiskers. The Wilcoxon signed rank test was applied. C. Correlation between SP and TP in evaluating PMN-MDSC frequency in *ex vivo* PBMC was analyzed by the Spearman test. A p-value <0.05 was considered statistically significant.

**Fig 3:**
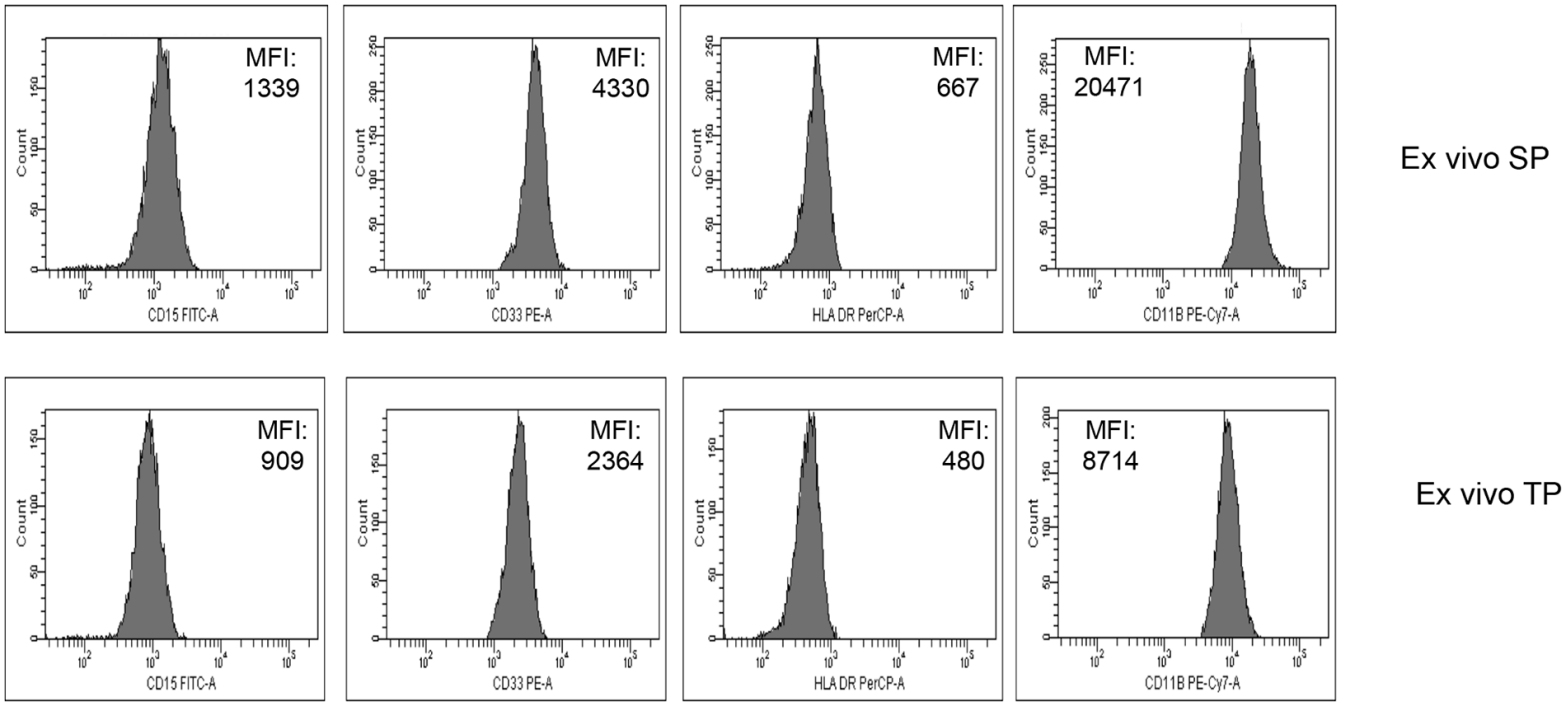
MFI of CD15, CD33, CD11b, and HLA-DR are lower using TP than SP. Representative histogram plots showing the MFI of CD15, CD33, CD11b, and HLA-DR after staining *ex vivo* PBMC with SP and TP. MFI values are indicated in the plots.

To verify if the TP could alter the identification of PMN-MDSC in *ex vivo* PBMC compared to SP, we first applied the SP and TP protocols to freshly isolated cells. PMN-MDSC in PBMC from HIV+ patients were analyzed by flow cytometry. Fig 2A shows representative plots. Comparing data obtained with the 2 protocols, we did not observe any significant difference (Fig 2B). Moreover, when we correlated the frequencies of PMN-MDSC obtained by SP and TP we obtained a rho (r) of 0.98 (p<0.0001), indicating that TP protocol does not alter the frequency of PMN-MDSC (Fig 2C). As expected, when comparing the mean fluorescence intensity (MFI) of markers using TP and SP we found that the MFI of CD15, CD33, CD11b, and HLA-DR were lower using TP than SP, suggesting that monoclonal antibodies bound their targets with less efficiency in TP than in SP (Fig 3), possibly due to cell fixation before staining. Yet, the lower MFI did not threaten the identification of the correct frequency of PMN-MDSC.

### TP allows the correct identification of PMN-MDSC frequency in cryopreserved samples

**Fig 4:**
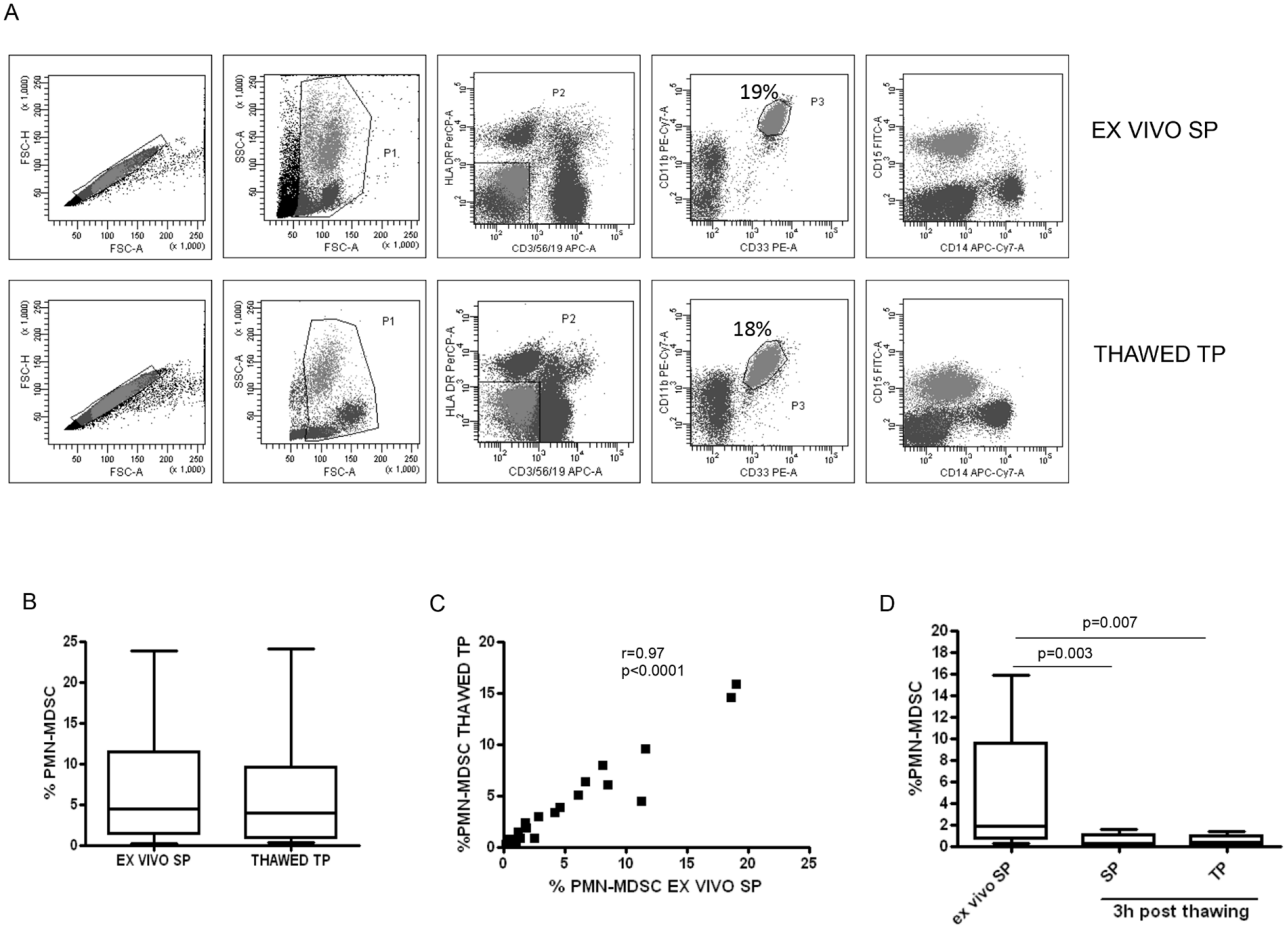
PMN-MDSC frequency from thawed PBMC evaluated with TP is comparable to that tested on *ex vivo* PBMC using SP. A. Representative dots plots showing the gating strategy to identify PMN-MDSC in *ex vivo* and thawed PBMC stained with SP and TP protocols, respectively: after doublets exclusion (FSC-A/FSC-H), in the morphological gate (FSC/SSC) we excluded debris, then we gated Lin^-^/HLA DR^low/-^ cells. In this gate we selected CD11b^+^CD33^+^ cells (MDSC). To discriminate PMN-MDSC and Mo-MDSC, the expression of CD14 and CD15 is shown on cells selected from the CD11b^+^CD33^+^ gate. B. PMN-MDSC frequency in *ex vivo* SP and thawed TP stained PBMC from 22 HIV infected patients. Results are shown as Box and Whiskers. The Wilcoxon signed rank test was applied. C. The correlation between PMN-MDSC frequency in *ex vivo* SP stained and thawed TP stained PBMC was evaluated by the Spearman test. D. PMN-MDSC frequency in *ex vivo* and 3 hours post thawing PBMC (n = 13) stained with SP and TP protocols. The Wilcoxon signed rank test was applied. A p-value <0.05 was considered statistically significant.

Then we test if TP could overcome the loss of PMN-MDSC in cryopreserved samples. In Fig 4A, an example of dot plots showing *ex vivo* cells stained with the SP and thawed cells stained with TP are reported. In particular, no significant difference of PMN-MDSC frequency between the two protocols was found (Fig 4B). Moreover, the correlation analysis showed a high conformity between the protocols (r = 0.97, p<0.0001) (Fig 4C), indicating that TP can be used to identify PMN-MDSC in cryopreserved samples without underrating cells. Differently, when PBMC were cultured for 3 hours after thawing, PMN-MDSC were not assessable anymore with both SP and TP (Fig 4D), confirming that PMN-MDSC are very susceptible to freezing and thawing thus making fixation soon after thawing indispensable to perform a phenotypic analysis. To note that, when TP was performed on thawed PBMC, the CD15^+^CD14^+^ PMN-MDSC were not present (Fig 4A), indicating that TP overcome the alteration of PMN-MDSC phenotype in cryopreserved samples observed using SP.

**Fig 5:**
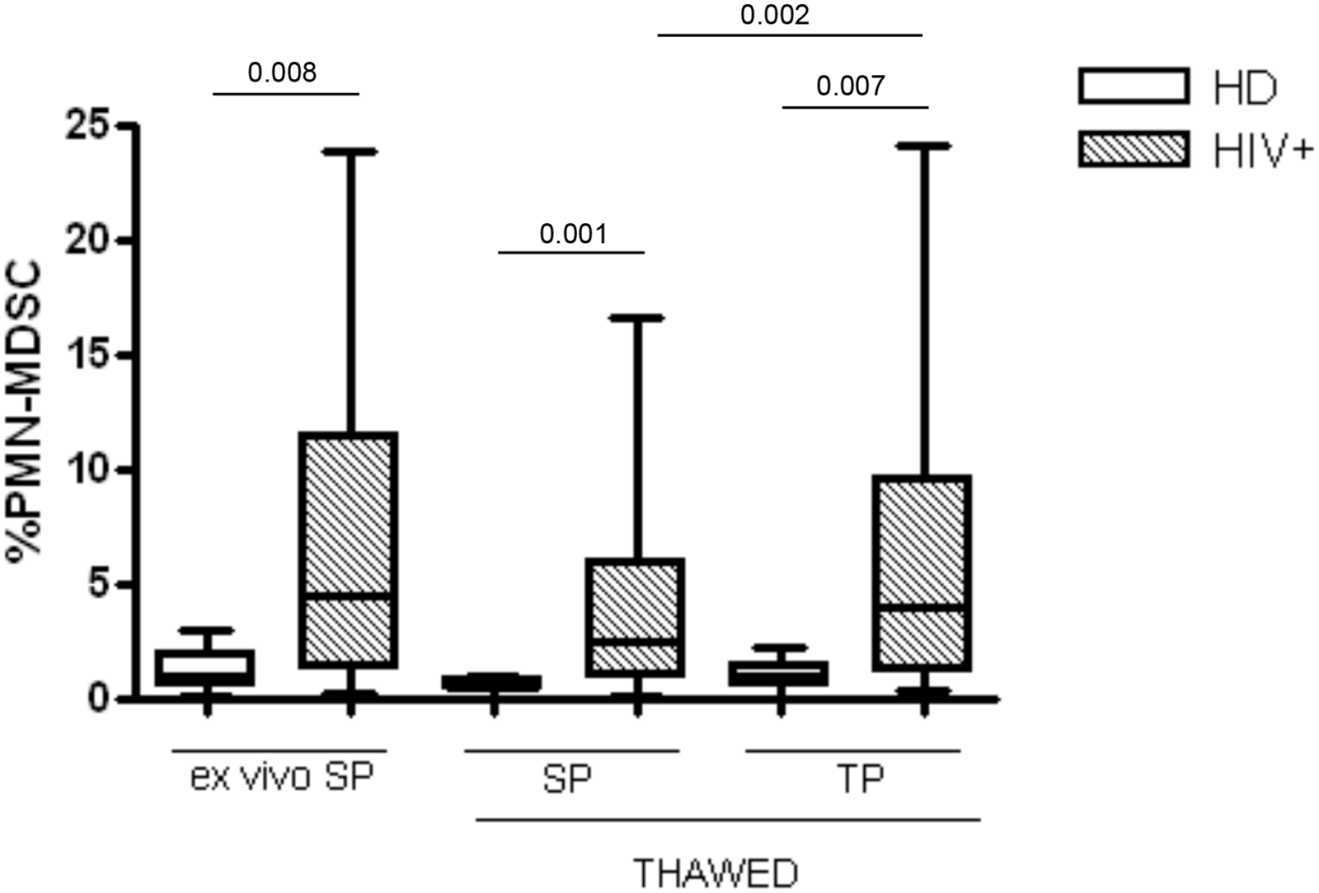
TP preserves the right difference in PMN-MDSC frequency between HD and HIV+ patients. PMN-MDSC frequency in HD (n = 16) and HIV+ patients (n = 22) evaluated in *ex vivo* PBMC stained with SP, and thawed PBMC stained with SP and TP. Results are shown as Box and Whiskers. The Mann Whitney test was applied. A p-value <0.05 was considered statistically significant.

We then analyzed the impact of TP and SP in evaluating the difference in PMN-MDSC frequency between HIV+ patients and healthy donors (HD). According with previous reports, we found that in HIV+ patients PMN-MDSC frequency analyzed in thawed PBMC stained with both SP or TP was statistically different from HD. However, the p value obtained using SP on thawed cells was lower than that using *ex vivo* PBMC (0.001 vs 0.007, Fig 5), probably because of the lower range of PMN-MDSC frequency in thawed PBMC from HD stained with SP (0%-0.9%) compared to *ex vivo* PBMC (0.1%-3%). Differently, the p value obtained using TP on thawed cells was comparable to that using SP on *ex vivo* PBMC (0.008 vs 0.007), confirming that TP is an accurate protocol to utilize on thawed cells. Finally, a significant difference between SP and TP performed on thawed PBMC was confirmed (Fig 5).

## Discussion

MDSC represents a heterogeneous cell population that play a significant role in the development of immune tolerance to cancer, infectious diseases, and autoimmunity [21–23]. For this reason, in future MDSC may be used as predictive marker in numerous cancers, and the possibility to deplete MDSC *in vivo* opens up to new therapeutic approaches [24].

Although cryopreservation of PBMC is a widely used procedure, it has been shown that it may alter the phenotype and function of peripheral blood mononuclear cells [25]. Further, some cell populations among PBMC are sensitive to freezing/thawing procedure. For example, Treg frequency was reported to be reduced after cryopreservation [26].

It has been shown that cryopreservation has an impact on the PMN-MDSC viability and recovery, which significantly alters such MDSC population [20]. However, in retrospective case-control studies or in research settings that envisage samples shipping, cryopreservation is often inevitable. Herein, we demonstrated that if thawed PBMC were immediately fixed in 1% formalin and then stained for PMN-MDSC identification, their frequency is comparable to that of *ex vivo* PBMC. On the contrary, 3 h after thawing PMN-MDSC percentage dramatically declines, suggesting that fixation immediately after thawing is essential to correctly estimate PMN-MDSC frequency. These results also confirm that, even if PMN-MDSC are recognizable when cells are immediately fixed after thawing, they are very susceptible to thawing, making functional studies not feasible. We previously demonstrated that T cell functions from HIV+ patients are restored soon after depletion of MDSC [9], thus PMN-MDSC loss after thawing could result in a misinterpretation of T cell capacities.

Our data indicate that, when comparing HD and HIV+ patients, SP and TP performed on cryopreserved cells soon after thawing are both capable to show the difference in the PMN-MDSC frequency between the two groups (even if with a different precision and accuracy). However, we also showed that the longer the time after thawing, the higher the PMN-MDSC loss. Then, we can speculate that the use of *ex vivo* or thawed cells in the evaluation PMN-MDSC may explain some controversial results that have been published about MDSC subsets expanded in HIV infection [8–10, 15].

A standard procedure able to correctly test PMN-MDSC in cryopreserved PBMC may be very useful to make results from differently manipulated samples more reproducible.

In conclusion, we demonstrated that fixing PBMC soon after thawing and before antibody staining allows preservation of PMN-MDSC integrity and cells quantification. Thus, it is possible to phenotipically identify PMN-MDSC in cryopreserved PBMC, consenting adequate test precision and accuracy as well as making multicentre research more feasible.
